# S100A4 (p9Ka) protein in colon carcinoma and liver metastases: association with carcinoma cells and T-lymphocytes

**DOI:** 10.1038/sj.bjc.6600071

**Published:** 2002-02-01

**Authors:** S Taylor, S Herrington, W Prime, P S Rudland, R Barraclough

**Affiliations:** Cancer Tissue Bank Research Centre, University of Liverpool, P.O. Box 147, Liverpool, L69 7ZB, UK; School of Biological Sciences, University of Liverpool, P.O. Box 147, Liverpool, L69 7ZB, UK

**Keywords:** p9Ka, S100A4, T-lymphocytes, colon carcinoma, metastasis

## Abstract

The presence of the EF-hand-calcium-binding protein S100A4 in the carcinoma cells of the primary tumour is associated with a shorter survival time of a group of breast cancer patients. In colon cancer, primary tumours as well as metastases to the liver can be studied. Here we show, using quantitative PCR applied to RNA from 24 normal colon, four liver tissues, 24 colon carcinoma specimens, and 24 livers containing colonic carcinoma metastases, that the level of S100A4 mRNA was significantly higher in the carcinomas compared to normal specimens (Mann-Whitney *U*-test, *P*=0.05), and in liver metastases compared to carcinoma specimens (*P*=0.039). The latter comparison included seven liver metastases and their matched primary carcinomas (*P*<0.001) from the same patient. *In situ* hybridization and immunocytochemistry techniques have localized S100A4 to both carcinoma cells and lymphocytes in the malignant specimens. The percentage of specimens stained for S100A4 in the epithelial cells is significantly higher for those isolated from carcinomas and metastases than from the corresponding normal tissue, and from metastases than from corresponding carcinoma (Fisher Exact text, *P*<0.0016, *P*=0.04, respectively). In most specimens, S100A4 is present in clusters of T lymphocytes and this distribution is also found in the lymphoid, uninflamed appendix.

*British Journal of Cancer* (2002) **86**, 409–416. DOI: 10.1038/sj/bjc/6600071 www.bjcancer.com

© 2002 The Cancer Research Campaign

## 

The EF-hand-containing protein, S100A4 (p9Ka) is closely linked with the ability of mammary tumour cells to metastasize in experimental model systems. Transfection of the gene for rat (Davies *et al*, 1993) or human (Lloyd *et al*, 1998) S100A4 into benign rat mammary cells, and the expression of S100A4 protein at an elevated level leads to the expression of a metastatic phenotype. Similar results have been obtained by over-expressing the mouse S100A4 in human MCF-7 cells (Grigorian *et al*, 1999). Mice, transgenic for the S100A4 gene, which over express S100A4, show no phenotype, but when these mice are mated with mice bearing, as an expressed transgene, an activated oncogene, such as c-*erb*B-2, which produces benign mammary tumours, offspring which express both the onco-transgene and the S100A4 transgene, exhibit tumours which also metastasize to the lungs (Davies *et al*, 1996). A similar result was obtained when transgenic mice bearing the mouse S100A4 transgene were mated with a strain of mice which yielded MMTV-induced mammary tumours (Ambartsumian *et al*, 1996).

In a study of 349 patients with breast cancer, the presence of immunocytochemically-detectable S100A4 in the primary tumours is associated with their early demise when followed up for 19 years (Platt-Higgins *et al*, 2000; Rudland *et al*, 2000). This result suggests a direct link, at least in this group of patients, between the presence of S100A4 in the carcinoma cells of the primary tumour and the process of metastasis, since it is commonly believed that breast cancer patients die of metastatic spread to sites other than that of the lymph nodes. Thus it is important to establish whether the cells which metastasize to these distant sites are the cells which contain high levels of S100A4. However, it is not possible to study easily, the distant metastases which arise from the primary tumour of breast cancer, since metastatic spread in breast cancer is predominantly to surgically inaccessible sites such as the bones and brain of the patients. In contrast the primary tumour of colon carcinomas metastasises at a relatively high frequency to the liver, and pathological tissue from liver containing such metastases is frequently surgically resected and is therefore available. Thus, the levels of S100A4 have been determined in the primary tumour of colon carcinomas and corresponding liver metastases using quantitative polymerase chain reaction, *in situ* hybridization and immunocytochemical techniques. The results suggest that some variability in the level of S100A4 in tissue specimens is due to infiltrating cells, such as T-lymphocytes, but that S100A4 is present at a higher level in the carcinoma cells of liver metastases derived from colon carcinomas than the corresponding primary colon carcinomas.

## MATERIALS AND METHODS

### Tissue specimens

Tissue specimens were obtained with ethical approval and full informed consent from patients treated at the Royal Liverpool University Hospital between 1993 and 2000. Primary colon carcinoma, adjacent normal colon from specimens resected for colon carcinoma, liver metastases, adjacent normal liver from specimens resected for cancer in the liver, and specimens of colon adenoma were snap frozen in liquid nitrogen within 1 h of surgery. The frozen tissue was stored at −140°C until processed for isolation of RNA. Adjacent samples were fixed in formalin prior to being embedded in paraffin wax. Six μm serial sections were cut for immunocytochemistry and *in situ* hybridization, the latter under RNase-free conditions (Taylor *et al*, 1999). The pathological details of the specimens studied are shown in Table 1

**Table 1:**
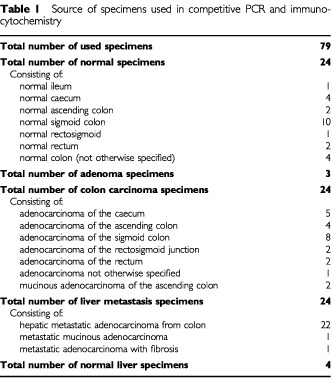
Source of specimens used in competitive PCR and immuno cytochemistry

. Normal appendix was obtained from a right hemicolectomy specimen removed for adenocarcinoma of the transverse colon.

### Immunocytochemistry

Sections of formalin-fixed paraffin-embedded material were dried overnight at 37°C, dewaxed in xylene and rehydrated through graded concentrations of ethanol. Peroxidases were blocked using 1% H_2_O_2_ in methanol for 12 min. Immunocytochemistry for S100A4 was carried out as described previously (Rudland *et al*, 2000). The primary S100A4 antibody (DAKO, Ely Cambs., UK) was used at a dilution of 1 : 400, and was incubated with the sections overnight. The secondary antibody, donkey anti-rabbit IgG, was used at a dilution of 1 : 300. Sections were counterstained with Mayers haemalum and mounted in DPX (BDH, Nottingham, UK).

To compare the localization of S100A4 within the two major types of lymphocytes, T and B cells, immunocytochemistry was performed on 5 μm serial sections of formalin-fixed, paraffin-embedded normal appendix. The antibodies to CD79a (B cells) and to CD3 (T-cells) were obtained from DAKO (Ely, Cambs., UK) and are specific for their respective subgroup of lymphocytes (Chetty and Gatter, 1994; Mason *et al*, 1995).

Immunocytochemistry for all three antibodies was performed with the Dako Envision horseradish peroxidase system using diaminobenzidine (DAB) as the chromogen. Prior to incubation of sections of appendix with the primary antibodies, they were heated in 10 mM EDTANa_2_ pH 7.0 in a pressure cooker at 10 p.s.i. for 2.5 min for retrieval of antigens. All washes between each stage were performed with Tris buffered saline (TBS) (50 mM Tris-HCl (pH 7.4), 150 mM NaCl).

Monoclonal antibodies to lymphocyte antigens were diluted 1 : 50 and antibodies to S100A4 were diluted 1 : 200 in 5% (w/v) bovine serum albumin in TBS (pH 7.4). They were applied to the tissue sections for 40 min at room temperature, after a pre-treatment of the sections with 5% (w/v) bovine serum albumin in TBS (pH 7.4) for 10 min for the polyclonal antiserum to S100A4. All subsequent steps followed the polyclonal and MAb protocols supplied with the Dako Envision+ HRP system. The appropriately labelled polymer was applied for 30 min, washed with buffer and the horseradish peroxidase was visualized with DAB/water solution supplied with the DAKO Envision kit. Sections were counterstained with Mayer's haemalum for 30 s and processed as above.

### Competitive RT*–*PCR for S100A4 mRNA

**Figure 1:**
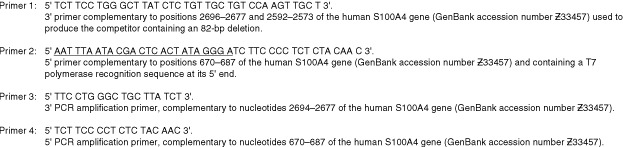
Primers used for competitive reverse-transcript polymerase chain reaction. The recognition sequence for T7 RNA polymerase, used to make the competitor RNA, is underlined.

Competitive RT–PCR to quantify S100A4 mRNA was based upon a previously-described method (Taylor *et al*, 1998) with the following modifications. Primers 1 and 2 (Figure 1

) were used in RT–PCR reactions to create a PCR product that contained a T7 transcription sequence. This PCR product coupled with T7 RNA polymerase produced an S100A4-specific competitor *RNA* that was 82 bp shorter than the target fragment of S100A4 mRNA. Both target and competitor S100A4 mRNA were amplified using primers 3 and 4 in the same reaction (Figure 1). The competitor *RNA* was quantified precisely by measuring its optical density at 260 nM, and 10 serial two-fold dilutions were prepared containing known concentrations of competitor RNA ranging from 20 000–39 pg μl^−1^. In the reverse-transcription reaction, 0.5 μg of primer 3 (Figure 1), a known amount of competitor RNA and 500 ng of target RNA were incubated at 56°C for 10 min before being placed on ice. The Superscript mixture (GIBCO–BRL, Paisley Scotland, UK) was added before being placed at 37°C for 1 h. The PCR reaction mixture contained 1×PCR buffer (GIBCO–BRL), 10 pmol each of primers 3 and 4 (Figure 1), 2 mM MgCl_2_, 0.02% (w/v) detergent W-1, 0.2 mM dNTPs and 1.5 units Taq DNA polymerase (GIBCO–BRL). The temperature cycling profile for amplification was: one step of 95°C for 5 min, followed by 30 cycles of 95°C for 45 s, 56°C for 45 s and 72°C extension for 45 s. A final step of 72°C for 10 min completed any unfinished extensions. Co-amplification of target and competitor with primers 3 and 4 produced fragments of 366 and 284 bp, respectively. RNA from one carcinoma specimen (sample 56) was included in each run of competitive PCR as a normalizing control.

### Preparation of RNA probes for* in situ* hybridization

A 334 bp fragment of the 3′ end of the human S100A4 cDNA consisting of part of exon 3 to the poly(A) addition sequence (position 6940–7274 of the DNA sequence, Genbank accession number Z33457) was amplified by PCR and cloned into the *Eco*RI and *Not*I restriction enzyme sites of pBluescript (Stratagene, Amsterdam, The Netherlands). Plasmid containing the S100A4 fragment was linearized with either restriction enzymes *Eco*RI or *Not*I (Roche, Lewes, UK) prior to transcription of antisense and sense RNA's from the T3 and T7 promoters, respectively, as described previously (Taylor *et al*, 1999). Transcripts were purified *in vitro* from template DNA, labelled with Digoxygenin (DIG) (Roche) and incorporation was quantified, as previously described (Taylor *et al*, 1999). RNA probes were stored in aliquots at −80°C until required.

### In situ *hybridization*

*In situ* hybridization was performed on histological sections as described previously (Taylor *et al*, 1999). Briefly, the sections were dewaxed and rehydrated before being treated with proteinase K (Sigma, New Orleans, USA) for 30 min at 37°C. Sections were post-fixed in 4% (w/v) paraformaldehyde, acetylated and prehybridized before being incubated with 100 ng slide^−1^ of the appropriate RNA sense or antisense probe in hybridization buffer (Taylor *et al*, 1999). Sections of each tissue specimen were incubated with antisense or sense probes. After a stringent washing procedure (Taylor *et al*, 1999), sections were blocked with blocking reagent (Roche) and then incubated with 1 : 400 dilution of anti-DIG-conjugated to alkaline phosphatase (FAB fragment, Roche), before the detection solution containing nitro blue tetrazolium salt (NBT), chloro-3-indolyl-phosphate (BCIP) and levamisole was added and the slides were left for 16 h in the dark in a Hybaid Omnislide unit for colour to develop at room temperature. Three independent observers assessed the resulting stained sections for the level of staining.

## RESULTS

**Figure 2:**
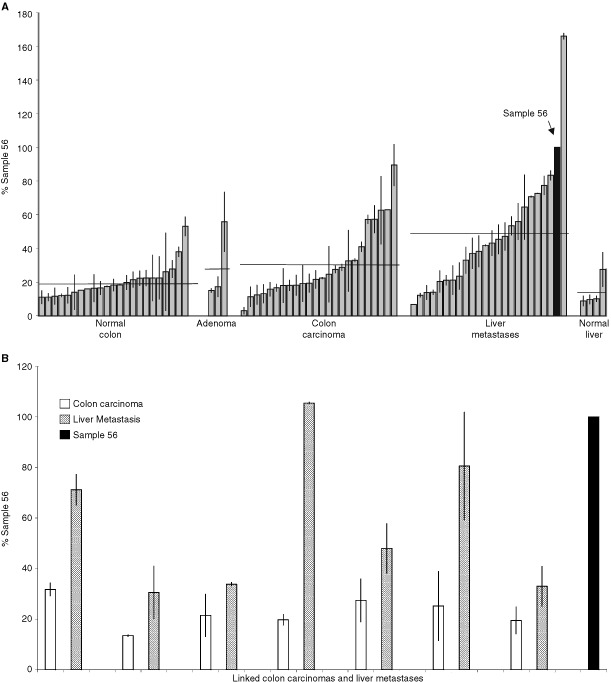
Competitive RT–PCR of S100A4 mRNA from tissue and tumour specimens. RNA isolated from specimens of (**A**) normal colon, benign colonic adenoma, colon carcinoma, liver with metastases, normal liver and (**B**) a sub-group of linked specimens of colon carcinomas and metastases in the same patients were subjected to quantitative RT–PCR and the level of S100A4 mRNA expressed as a percentage of that of one of the specimens (specimen 56) which was included in each experiment to allow comparison of samples analyzed in different experiments. The error bars represent ±s.d. of two experiments. The mean level for each group of specimens is shown by the horizontal lines in **A**.

**Figure 3:**
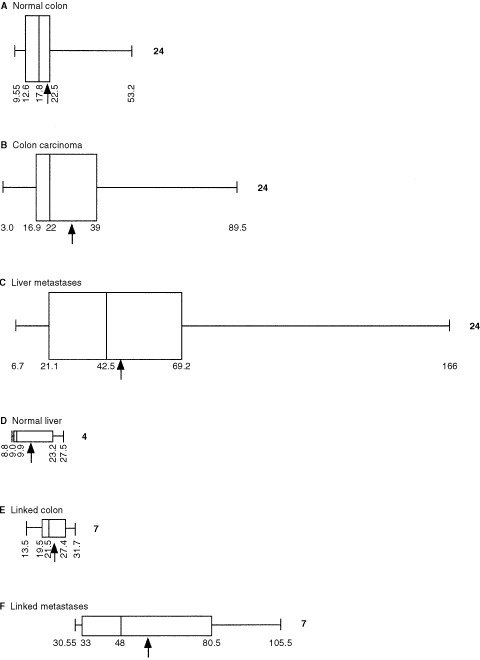
Box and Whisker plot of S100A4 mRNA levels in normal tissue and tumour specimens by quantitative competitive PCR. RNA isolated for tissue specimens was subjected to quantitative competitor PCR and the levels of S100A4 mRNA determined as a per cent of the control specimen, number 56. The median, upper and lower quartile, maximum and minimum value of the levels of S100A4 mRNA are shown as numbers beneath each Box and Whisker plot for specimens of (**A**) normal colon, (**B**) colon carcinoma, (**C**) liver metastases derived from colon carcinoma, (**D**) normal liver, (**E**) linked carcinoma and (**F**) linked liver metastases. The arrows below the boxes point to the values of the means. The width of the boxes represents the number of specimens in each group, which is also shown in bold alongside each plot.

The levels of S100A4 mRNA in RNA isolated from 24 specimens each of normal colon, colon carcinoma and liver metastases derived from colon carcinomas, three specimens of benign adenoma of the colon, and four specimens of normal liver were determined by quantitative competitor RT–PCR, as described in Materials and Methods (Figure 2A

). Amounts of S100A4 mRNA/500 ng of total RNA from each tissue were expressed as a percentage relative to one specimen of colon carcinoma (labelled Sample 56 in Figure 2). Generally low levels of S100A4 mRNA, relative to the control specimen 56, were evident in the normal tissue specimens from colon (mean=19.8%±s.e.2.0) and liver (mean=14%±s.e. 4.5). However, higher mean levels of S100A4 mRNA in the RNA from the specimens of colon carcinomas (mean=30%±s.e. 4.3) and liver metastases from colon carcinoma patients (mean=49%±s.e. 7.2) were clearly evident over those in the normal tissues. A preliminary analysis of variance using the Kruskal–Wallace Test indicated that colon carcinoma *vs* normal liver, colon carcinoma *vs* normal colon, liver metastases *vs* normal liver, liver metastases *vs* normal colon, and liver metastases *vs* colon carcinomas were likely to be statistically significantly different. The statistically significant differences were confirmed using a two-tailed Mann–Whitney *U*-Test (*P* values, respectively=0.042, 0.05, 0.012, 0.0003, 0.039) and the results are shown as Box and Whisker plots (Figure 3A–D

). An additional set of specimens from seven patients was available consisting of samples of colon carcinoma and of liver metastases from the same individual. These paired specimens of primary carcinoma and secondary liver metastases exhibited mean values of S100A4 mRNA of 22.7%±s.e. 2.6 and 57.5%±s.e. 10.9 respectively. These values represented a statistically-significant higher level of S100A4 mRNA in the liver metastases than in the primary cancer specimens (Figures 2B and 3E,F) when analyzed either as two groups (*P*<0.001, two-tailed Mann–Whitney *U*-Test) or as matched pairs (*P*<0.015, Wilcoxon Signed Rank Pairs test). When the colon carcinomas were subdivided according to the Duke's classification, the S100A4 mRNA levels for class A (non invasive), class B (invasive with no nodal involvement), class C (metastasis to regional lymph nodes) were not significantly different (A *vs* B, *P*=0.933; A *vs* C, *P*=0.888; B *vs* C, *P*=0.11; 2-tailed Mann–Whitney *U*-Test.

**Figure 4:**
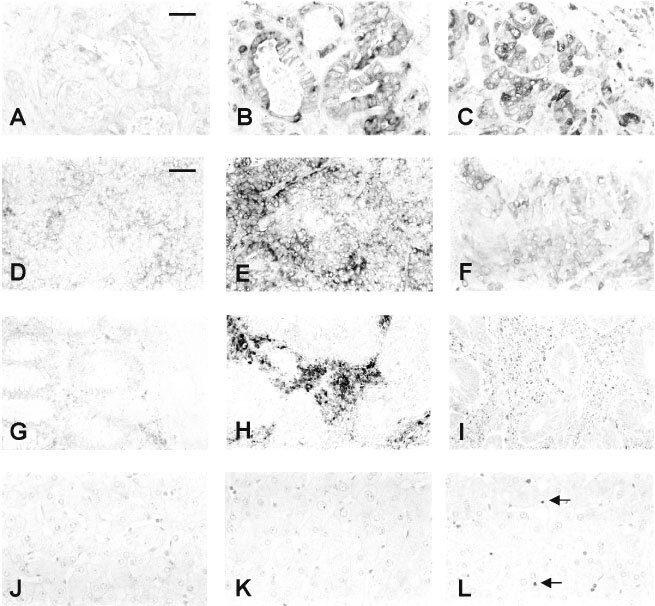
*In situ* hybridization and immunocytochemical staining of tissue and tumour specimens. Histological sections of specimens of liver containing metastases (**A**–**C**), of primary colon carcinoma (**D**–**I**), and normal liver (**J**–**L**) were subjected to *in situ* hybridization (**A**,**B**,**D**,**E**,**G**,**H**,**J**,**K**) with sense (**A**,**D**,**G**,**J**) or antisense (**B**,**E**,**H**,**K**) probes corresponding to S100A4 mRNA, or subjected to immunocytochemistry with anti-S100A4 serum (**C**,**F**,**I**,**L**) as described in Materials and Methods. Following development of colour, images of the sections were digitized and printed. Arrows show stained lymphocytes. Magnification ×144 (**A**–**C**, **J**–**L**), ×72 (**D**–**I**); bar on **A**=50 μm, bar on **D**=100 μm.

In order to identify the source of the S100A4 in the tissue specimens, histological sections of the specimens were subjected to *in situ* hybridization using sense and anti-sense probes for S100A4 mRNA, as described in Materials and Methods. There was very little S100A4 mRNA in normal liver (Figure 4J,K

), however, immunocytochemical staining of sections identified S100A4 protein in scattered cells (Figure 4L) which had the appearance of lymphocytes (arrows). However, in liver which contained metastases, there was strong staining of the carcinoma cells for S100A4 mRNA with the antisense probe (Figure 4B), but not with the sense probe (Figure 4A), under the same conditions. The presence of S100A4 in the carcinoma cells in the liver was confirmed by immunocytochemical staining with an antibody to S100A4 (Figure 4C). When *in situ* hybridization was carried out on some specimens of primary colon carcinoma which were positive for S100A4 mRNA in the quantitative RT–PCR assay, staining for this mRNA was evident in the carcinoma cells when the antisense probe (Figure 4E), but not the sense probe (Figure 4D) was used. The presence of S100A4 protein in the carcinoma cells was confirmed by immunocytochemical staining (Figure 4F). However, *in situ* hybridization revealed that in some other specimens of colon carcinoma which were positive in the quantitative RT–PCR assay, S100A4 mRNA appeared not to be generally present in the carcinoma cells (Figure 4G,H). This staining together with immunocytochemical staining of such specimens, localized the S100A4 mRNA/protein to clusters of small cells in the cancer specimen, which had the histological appearance of lymphocytes (Figure 4H,I). In the light of this finding, histological sections of all the specimens of normal colon, adenoma, colon carcinoma, normal liver and metastatic tumours in the liver were subjected to immunocytochemical staining for S100A4, and the presence of the S100A4 protein in epithelial cells and lymphocytes was assessed (Table 2

**Table 2:**
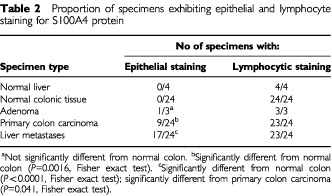
Proportion of specimens exhibiting epithelial and lymphocyte staining for S100A4 protein

). Of the 24 specimens of normal colon and four specimens of normal liver, all detectable S100A4 immunocytochemical staining was present in lymphocytes. All three of the adenomatous colon specimens exhibited lymphocyte staining with S100A4 being additionally present in the epithelial cells in one of the specimens. Nine of the 24 colon carcinoma specimens exhibited significant staining of the carcinoma cells, with three at a high level, and only in one of these specimens did the lymphocytes fail to stain for S100A4. Moreover 17 of the specimens of liver metastases also exhibited significant staining of the carcinoma cells (Fisher exact test, *P*<0.0001), however, only in one of the carcinoma specimens did the lymphocytes fail to stain for S100A4 (Table 2).

**Figure 5:**
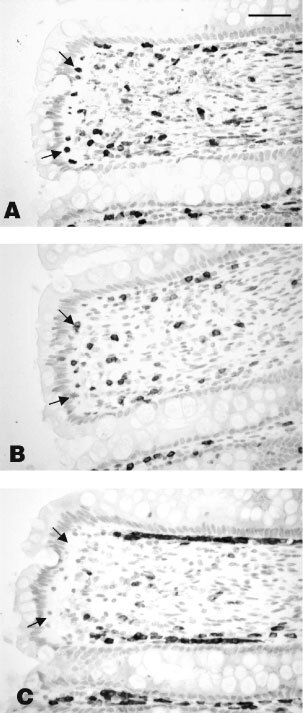
Immunocytochemical staining for (**A**) S100A4, (**B**) CD3 and (**C**) CD79a antigen in serial sections of the lamina propria of normal appendix. Staining for S100A4 can be seen to mirror that for CD3 (T cell marker) but not the pattern of CD79a (B cell marker). Arrows point to lymphocytes in the same regions on the serial sections. Magnification ×223, bar=50 μm.

In order to find out whether a particular subtype of lymphocytes contained the S100A4, serial sections of some tumour specimens were immunocytochemically stained with antibodies to S100A4, and also with antibodies to CD3 and CD79a antigens for T and B lymphocytes, respectively. S100A4 appeared to be associated with T lymphocytes (not shown), but the close association of T and B lymphocytes in the tumours prevented a firm conclusion to be drawn. Thus, serial sections of normal specimens of a human tissue with a characteristic distribution of T and B lymphocytes, namely human appendix, were similarly stained. S100A4 clearly co-localized to the region of the appendix containing predominantly immunocytochemically-determined T cells but not to the region containing predominantly immunocytochemically-determined B cells (Figure 5

arrows). Immunocytochemical staining for S100A4 (Figure 5A) was associated with individual cells staining for CD3 antigen (T lymphocytes) (Figure 5B), but not with cells staining for CD79a antigen (B lymphocytes) (Figure 5C).

## DISCUSSION

S100A4 has been shown to cause metastasis in rodent models of breast cancer (Davies *et al*, 1993, 1996; Ambartsumian *et al*, 1996), and its presence in breast cancer cells is strongly associated with death of a group of patients with human breast cancer (Platt-Higgins *et al*, 2000; Rudland *et al*, 2000). Using quantitative RT–PCR and *in situ* hybridization, S100A4 mRNA is found to be present in normal human colon and liver at only low levels. This result is in agreement with the previous finding that immunocytochemically-detected S100A4 is present at only a low level in colon and liver in the rat (Gibbs *et al*, 1995). Using competitive RT–PCR, the levels of S100A4 mRNA increase successively and significantly between normal tissue, carcinoma and liver metastases, and in every single matched case, the level of S100A4 mRNA is significantly higher in the liver metastases than the primary carcinoma. However using *in situ* hybridization techniques, S100A4 mRNA is found associated with both the colon carcinoma cells as well as other non-parenchymal cell types including lymphocytes, which suggests that both these cell types are synthesizing S100A4. Using immunocytochemistry, these results are broadly confirmed, the proportion of specimens with epithelial cells staining for S100A4 increases successively between normal tissue, carcinoma and liver metastases. In contrast, staining of lymphocytes for S100A4 remained at a consistently high value across the spectrum of tissues, in agreement with previous work in the breast (Nikitenko *et al*, 2000). Since there was no significant differences between the levels of S100A4 mRNA in different Duke subtypes of colon carcinoma, it is possible that expression of higher levels of S100A4 are required more for dissemination to distant metastatic sites than for local invasion and dissemination to regional lymph nodes. This idea is supported by immunocytochemical experiments (Bronckart *et al*, 2001) in which there was no statistically-significant difference between normal colon and non-metastatic colon carcinoma specimens when the proportion of specimens staining for S100A4 was examined. However, in the same study (Bronckart *et al*, 2001), it was found that the percentage of epithelial or connective tissue cells staining for S100A4 decreased with increasing malignancy grade amongst dysplastic lesions and carcinomas. That this trend was not observed in the present experiments is likely to be due to the small number of benign lesions available.

Our results are broadly consistent with those obtained previously (Takenaga *et al*, 1997) where a similar increase in immunocytochemically-detected S100A4 was observed in malignant relative to non-malignant colonic tumour specimens. Moreover the deeper, more invasive regions of the specimens in that study were enhanced in immunocytochemically detectable S100A4, suggesting an association of higher levels of S100A4 protein with the invading regions of the tumours (Takenaga *et al*, 1997). However, in the present experiments, the proportions of specimens exhibiting positive staining of the epithelial cells for S100A4 were somewhat different from those in the previous study, 33% of adenomas positive (0% positive, previously), 38% of adenocarcinoma specimens positive (94% positive, previously), 71% of liver metastases positive (100% positive, previously). Whilst the small number (three) of adenoma specimens used in the present study may have contributed to the different results with this group of specimens, this is unlikely to be the explanation for the carcinomas and liver metastases, since 24 specimens of each were used. It is possible that the quantitative differences between the two studies may have arisen from differences in the nature of the specimens used.

Recently, in a separate study, S100A4 levels as detected by Western blotting, were found to be higher in seven of 12 colon adenocarcinoma specimens than in adjacent normal tissue. However, the result was found not to be statistically significant (Komatsu *et al*, 2001). Whilst it is possible that S100A4 protein was also elevated in the adjacent normal tissue, it is more likely that this result arises from variability in the levels of S100A4 in the carcinoma specimens as measured by Western blotting, due to the presence of S1004 in infiltrating lymphocytes, as found in the present study. When the S100A4 levels in epithelial cells were compared immunocytochemically (Table 2), the proportion of specimens exhibiting epithelial staining for S100A4 was more strongly significantly different between carcinomas and normal colon (*P*=0.0016, Fisher exact test) than when specimens were compared using the quantitative RT–PCR technique (*P*=0.05, Fisher exact test). The quantitative RT–PCR technique, like the Western blotting used by Komatsu *et al* (2001) does not distinguish between staining of epithelial and infiltrating host cells.

The present observation that all specimens contain immunocytochemical staining of lymphocytic cells for S100A4 is consistent with previous reports that S100A4 is found in human cells cultured from the immune system, the promyelocytic leukaemia cell line, HL-60 (Takenaga *et al*, 1994), the acute lymphoblastic leukaemia cell line, MOLT-4, and human peripheral lymphocytes treated with interleukin 2 (Tulchinsky *et al*, 1995), as well as in lymphocytes associated with human breast cancer (Nikitenko *et al*, 2000; Rudland *et al*, 2000). S100A4 is found in the immune cells of the appendix, where it localizes to the T, and not the B lymphocytes. Whilst this specimen of appendix was of normal appearance, it had been removed from a colon cancer patient, but had been located at least 45 cm from the carcinoma in the gut. Thus, the possibility cannot be entirely eliminated that the appearance of S100A4 in the T cells of the appendix might have been, in some way affected by the presence of the carcinoma cells in the gut. The present experiments show, for the first time, using markers for T and B lymphocytes, that S100A4 is found exclusively in T lymphocytes, not only in the immune cells of the appendix, but also in specimens of colon carcinoma. The results suggest that S100A4 may also have a normal role in cytotoxic immune reactions as well as in metastasis of carcinoma cells.
